# Targeting bioenergetics is key to counteracting the drug-tolerant state of biofilm-grown bacteria

**DOI:** 10.1371/journal.ppat.1009126

**Published:** 2020-12-22

**Authors:** Monique Donnert, Sarah Elsheikh, Alejandro Arce-Rodriguez, Vinay Pawar, Peter Braubach, Danny Jonigk, Axel Haverich, Siegfried Weiss, Mathias Müsken, Susanne Häussler

**Affiliations:** 1 Institute of Molecular Bacteriology, TWINCORE, Centre for Experimental and Clinical Infection Research, Hannover, Germany; 2 Department of Molecular Bacteriology, Helmholtz Centre for Infection Research, Braunschweig, Germany; 3 Department of Molecular Immunology, Helmholtz Centre for Infection Research, Braunschweig, Germany; 4 Institute of Immunology, Hannover Medical School, Hannover, Germany; 5 Institute of Pathology, Hannover Medical School, Hannover, Germany; 6 Biomedical Research in Endstage and Obstructive Lung Disease Hannover (BREATH), German Center for Lung Research (Deutsches Zentrum für Lungenforschung [DZL]), Hannover, Germany; 7 Department of Cardiothoracic, Transplant and Vascular Surgery, Hannover Medical School, Hannover, Germany; 8 Central Facility for Microscopy, Helmholtz Centre for Infection Research, Braunschweig, Germany; 9 Department of Clinical Microbiology, Copenhagen University Hospital–Rigshospitalet, Copenhagen, Denmark; 10 Cluster of Excellence RESIST (EXC 2155), Hannover Medical School, Hannover, Germany; university of washington, UNITED STATES

## Abstract

Embedded in an extracellular matrix, biofilm-residing bacteria are protected from diverse physicochemical insults. In accordance, in the human host the general recalcitrance of biofilm-grown bacteria hinders successful eradication of chronic, biofilm-associated infections. In this study, we demonstrate that upon addition of promethazine, an FDA approved drug, antibiotic tolerance of *in vitro* biofilm-grown bacteria can be abolished. We show that following the addition of promethazine, diverse antibiotics are capable of efficiently killing biofilm-residing cells at minimal inhibitory concentrations. Synergistic effects could also be observed in a murine *in vivo* model system. PMZ was shown to increase membrane potential and interfere with bacterial respiration. Of note, antibiotic killing activity was elevated when PMZ was added to cells grown under environmental conditions that induce low intracellular proton levels. Our results imply that biofilm-grown bacteria avoid antibiotic killing and become tolerant by counteracting intracellular alkalization through the adaptation of metabolic and transport functions. Abrogation of antibiotic tolerance by interfering with the cell’s bioenergetics promises to pave the way for successful eradication of biofilm-associated infections. Repurposing promethazine as a biofilm-sensitizing drug has the potential to accelerate the introduction of new treatments for recalcitrant, biofilm-associated infections into the clinic.

## Author summary

At sub-minimal inhibitory concentrations, phenothiazines have been shown to inhibit virulence as well as the formation of biofilms in a wide range of different bacterial pathogens. In this study, we analyzed the anti-bacterial effect of the FDA-approved drug, promethazine, on biofilm-grown *Pseudomonas aeruginosa*. We demonstrate that PMZ interferes with bacterial bioenergetics and sensitizes biofilm-grown *P*. *aeruginosa* cells to bactericidal activity of several different classes of antibiotics by several orders of magnitude. This effect was most pronounced when cells were grown under environmental conditions that induce low intracellular proton levels. Thus, it seems that a reduced proton efflux in cells that exhibit decreased respiratory activity due to their biofilm mode of growth might explain their general antimicrobial tolerance. The use of PMZ as an antibiotic sensitizer holds promise that targeting tolerance mechanisms of biofilm-grown bacteria could become a practicable way to change the way physicians treat biofilm-associated infections.

## Introduction

Biofilms are widely found in natural habitats and are considered an adaptation of microbes to hostile environments [1]. Bacteria in biofilms are embedded in a self-produced extracellular matrix and exhibit recalcitrance to a wide range of adverse conditions [2–4]. In the human host, biofilm-associated bacteria efficiently withstand antibiotic treatment and effectors of the immune system. Even in the absence of genotypic drug-resistance, antimicrobials have been demonstrated to be unable to clear biofilm infections [5–7]. Several factors, individually, or combinatorially, help to explain biofilm survival [8–12]. These include protection by extracellular polymeric substances (EPSs) [13,14], decreased antibiotic target activity due to decreased growth rate [15], biofilm-specific expression of possible resistance genes or gene products [16–18], and profound metabolic changes of biofilm-associated bacteria [19,20].

*Pseudomonas aeruginosa* is an exceedingly problematic gram-negative, opportunistic pathogen that has emerged as one of the most important human pathogens due to its role in acute nosocomial infections. Furthermore, chronic biofilm-associated *P*. *aeruginosa* infections, like those of the respiratory tract of cystic fibrosis patients, largely determine morbidity and mortality in the affected patients [21,22]. The urgent medical need for new therapy options spurred global biofilm research. However, despite a large body of research into biofilms, much remains to be learned about the mechanisms of biofilm formation and antimicrobial tolerance. Several anti-biofilm compounds have been described, with most interfering with structural biofilm components [6,23,24]. Potential anti-biofilm targets include adhesion molecules and biofilm matrix components such as extracellular DNA [25], lipopolysaccharides and exopolysaccharides [26]. Additionally, therapeutics that interfere with bacterial quorum sensing pathways [27,28] or secondary messengers involved in various signaling pathways [29–31] seem promising. Nevertheless, although there are numerous different biofilm targets, there are currently no anti-biofilm compounds in clinical use.

In this study, we analyzed the anti-bacterial effect of the phenothiazine promethazine (PMZ) on biofilm-grown *P*. *aeruginosa*. Phenothiazines are a class of highly versatile biological compounds and are well-known for their antipsychotic, sedative and antihistaminic effects [32]. Research conducted in the last couple of decades has indicated that phenothiazine compounds could play an important role in other fields of medicine as well, such as in the treatment of tumorous, neurodegenerative, or infectious diseases [33–35]. At sub-minimal inhibitory concentrations (MIC), phenothiazines have been shown to inhibit virulence as well as the formation of biofilms in a wide range of different bacterial pathogens [36–45].

Among the phenothiazines, the structure of promethazine (PMZ) is most similar to that of histamine. PMZ inhibits histamine H1 receptors and has an additional anticholinergic and a mild anti-dopaminergic activity. In this study, we show that PMZ interferes with bacterial bioenergetics and abrogates biofilm tolerance. PMZ sensitizes biofilm-grown *P*. *aeruginosa* cells to bactericidal activity of several different classes of antibiotics by several orders of magnitude. We also provide evidence for *in vivo* activity. The use of PMZ as an antibiotic sensitizer that targets microbial bioenergetics could change the way physicians treat chronically infected patients and pave the way for successful eradication of biofilm-associated infections.

## Materials and methods

### Ethics statement

All animal experiments were performed according to the guidelines of the German Recommendation of the Society for Laboratory Animal Science (GV-SOLAS) and the European Health Recommendations of the Federation of Laboratory Animal Science Associations. The animal protocol was approved by the local ethics committee and the “Niedersächsisches Landesamt für Verbraucherschutz und Lebensmittelsicherheit (LAVES)”, permission number 33.9-42502-04-12/0713.

### Bacterial strains, media and growth conditions

All *P*. *aeruginosa* strains and mutants used in this study, including the two reference strains PA14 and PAO1, are listed in S1 Table. Bacteria were cultivated in standard Lysogenic broth (LB) medium at 37°C with shaking (180 rpm) unless otherwise stated. For the cultivation of transposon mutants, 15 μg/ml gentamicin was added to the medium. Respiratory activity was measured following cultivation of the bacteria in a modified M9 minimal medium adapted from Abril *et al*. [46]. This medium contained 1 mM MgSO_4_, 0.1 mM CaCl_2_, 20 mM glucose, 0.01 mM FeSO_4_, 22 mM KCl, 5 mM HEPES, 2 mM Na_2_HPO_4_, 18.7 mM NH_4_Cl, 8.6 mM NaCl, and trace metals [H_3_BO_3_ 9.6 μM, ZnSo_4_ 0.7 μM, MnSO_4_ 0.45 μM, CoCl_2_ 2.1 μM, CuSO_4_ 0.15 μM, Na2MoO_4_ 0.3 μM]. Promethazine (PMZ) was used as a ready-to-use Promethazine hydrochloride injection solution (25 mg/ml, Promethazin-neuraxpharm, Neuraxpharm, Langenfeld, Germany).

To maintain the pH of the LB medium at 5.5, 7.2, and 8.5, respectively, we used the following buffer solutions: MES (2-(N-morpholino)ethanesulfonic acid), MOPS (3-(N-morpholino)propanesulfonic acid) and TAPS (N-[Tris(hydroxymethyl)methyl]-3-aminopropanesulfonic acid) at a final concentration of 50 mM. The buffers were prepared as 0.5 M stock solutions, adjusted to pH with NaOH/KOH at 37°C and filter-sterilized before use.

### Planktonic growth

Growth of planktonic *P*. *aeruginosa* PA14 cells with and without 1 μM, 10 μM, 25 μM and 100 μM PMZ was monitored using an automated growth analysis system (Bioscreen C MBR plate reader; Oy Growth Curves Ab, Helsinki, Finland). Overnight grown pre-cultures were diluted to a starting OD_600_ of 0.02 in 200 μl and the OD_600_ was measured every 30 minutes for up to 20 h.

### Bacterial respiration

Oxygen consumption rates (OCRs) and extracellular acidification rates (ECARs) of *P*. *aeruginosa* PAO1 and PA14 wild type strains were quantified using the Seahorse XFe96 Analyzer (Agilent, Santa Clara, California, USA). The protocol previously described by Dwyer *et al*. [47] was adopted and slightly modified. Briefly, overnight cultures of bacteria were grown in LB, diluted 1:100 in fresh modified M9 medium and grown to an OD_600_ of ~0.5 with shaking at 300 rpm. The cells were diluted to two times the final OD_600_ (OD_600_ of 0.05 for PA14 and OD_600_ of 0.04 for PAO1) in modified M9 medium. 90 μl were seeded onto poly-D-lysine-coated 96-well XF cell culture microplates. Cells were centrifuged for 10 min at 4,000 rpm in Heraeus Megafuge 40R centrifuge (ThermoFisher Scientific, Waltham, Massachusetts, USA), followed by increasing the well volume to 180 μl using modified M9 medium. Basal OCR and ECAR were measured for an initial 16 minutes, followed by automatic injection of 20 μl PMZ from 10x stock solutions (final concentrations of 200, 300 and 400 μM PMZ, respectively). OCR and ECAR measurements were then acquired every 4 minutes for up to 90 min. PMZ added to cell-free medium served as a control to account for background values upon PMZ addition; these OCR and ECAR values were subtracted to obtain final OCR and ECAR cell response profiles.

### Membrane potential

The *Bac*Light Bacterial Membrane potential kit (Thermo Fisher Scientific, Waltham, Massachusetts, USA) was used according to the manufacturer’s instructions. In brief, overnight grown cells were inoculated in 10 ml of LB medium at OD_600_ of ~0.05 and cultivated at 37°C until they reached early logarithmic phase (OD_600_ ~ 0.3–0.4). PMZ was added to the cultures at final concentrations of 100 μM, 250 μM and/or 400 μM, and the samples were incubated for an additional 10 minutes at 37°C. After PMZ addition, sample aliquots were directly diluted into 0.5 ml of filtered PBS (0.22 nm pore size) to approximately 10^6^ cells ml^-1^. The samples were exposed to 30 μM DiOC_2_(3) (3,3'-Diethyloxacarbocyanine Iodide) prepared from a 3 mM stock solution in DMSO, followed by further incubation for 30 min at room temperature in the absence of light. Stained bacteria were assayed in an LSR Fortessa flow cytometer (BD Biosciences, San Jose, California, USA) equipped with a 488 nm laser excitation source and both green (525/50 nm bandpass filter) and red (685/35 bandpass filter) fluorescence emission channels. F*orward* and side *scatter* density plots were used to identify the bacterial cell population of interest and to exclude *debris*. Fluorescence was recorded for at least 10,000 bacteria in both green and red emission channels and the red/green fluorescence ratios were subsequently calculated using population mean fluorescence intensities. The experiments were performed in independent triplicates.

### Biofilm microscopy

A static microtiter plate assay combined with automated confocal laser scanning microscopy was used as previously described [48]. Briefly, *P*. *aeruginosa* PA14 pre-cultures were grown overnight for 16 h in 3 ml of LB medium. One milliliter of each pre-culture was harvested by centrifugation (5000 g, 5 min), and the bacteria were washed twice with 1 ml of fresh LB. Cells were suspended in fresh LB medium, adjusted to OD_600_ of 0.002 and 100 μl of the bacterial solution were added to the wells of a sterile black half-area 96-well μClear microtiter plate (Greiner Bio-One). The microtiter plate was sealed with an air-permeable BREATHseal cover foil (Greiner Bio-One) and incubated for 24 h in a humid atmosphere at 37°C. The bacteria were then treated with PMZ at a concentration of 100 μM and stained by carefully adding the fluorescence dyes Syto9 and propidium iodide [final concentrations of 2.1 μM and 12.5 μM respectively] of the LIVE/DEAD BacLight Bacterial Viability Kit (Molecular Probes, Life Technologies) and incubated for another 24 hours.

Automated confocal microscopy was performed with an inverted SP8 system (Leica Microsystems) and the Leica application suite LAS X including the Matrixscreener module. Focal planes were acquired starting from the bottom of the plate (20 focal planes, z-step size: 3 μm) by using a PL APO 40x/1.10 W water immersion objective. Two Z-stacks (overview and zoom) were recorded in parallel at the center of a well: Stack 1 (overview stack) was acquired using a zoom x0.75, while stack 2 (zoom stack) was acquired with a zoom x4 in order to visualize biofilm details with higher resolution. Syto9 was excited at 488 nm, whereas propidium iodide (PI) was excited at 561 nm. Emission was detected with hybrid photo-detectors (HyD) in the range of 500–550 nm (Syto9) and 675–725 nm (PI) respectively. For automated image acquisition of all test samples, a number of pre-defined laser/detector settings were assigned to compensate for inter-well fluctuations in fluorescence intensity, avoiding under- and over-exposed images for the different treatments.

The image stacks were subsequently processed and analyzed with the software Developer XD 64 (Definiens) with a customized programmed solution. Moreover, biofilm structures were visualized with the software Imaris (version 7.6, Bitplane).

### Drug susceptibility testing and MIC determination

We performed antimicrobial susceptibility testing for tobramycin (TOB), ciprofloxacin (CIP), ceftazidime (CAZ) and gentamycin (GEN). Minimal inhibitory concentrations (MIC) were determined by a standard broth microdilution procedure [49] in LB medium using 96-well microtitre plates. Growth was evaluated after overnight incubation at 37°C with shaking at 180 rpm. The MIC was defined as the lowest antibiotic concentration for which no visible growth could be detected.

Antimicrobial susceptibility testing of biofilm grown bacterial cells was performed in a static microtiter plate assay as previously described [48] with slight modifications. In brief, overnight cultures of the cells were adjusted to an OD_600_ of 0.002 and 100 μl of the bacterial suspension were added to the wells of a sterile half-area, black 96-well μClear microtiter plate (Greiner Bio-One). The plate was sealed with an air-permeable BREATHseal cover foil (Greiner Bio-One) and incubated without shaking at 37°C in a humid atmosphere. After 24 h, the biofilms were treated with the antibiotics (at the given concentrations) either alone or in combination with PMZ (100μM) and in selected experiments also with 300 mM KCl, 20 μM CCCP (Carbonyl cyanide 3-chlorophenylhydrazone; Sigma Aldrich dissolved in DSMO) or 100 μM PQS (2-Heptyl-3-hydroxy-4(1H)-quinolone; Sigma Aldrich dissolved in DMSO).

The biofilms were incubated for another 24 h before the wells were resuspended and tenfold serial dilutions (10^−1^ to 10^−6^) were prepared using a 96-channel pipetting device (Platemaster, Gilson). Dilutions were spot-plated onto rectangular LB agar plates and incubated at 37°C overnight. Growth of surviving bacteria was evaluated after 16 h and colony-forming units (CFU) per ml were determined.

### Susceptibility on *ex vivo* lung tissue

In this study, tissue samples were obtained from three explanted lungs of CF patients undergoing double lung transplantation at Hannover Medical School (MHH). Procession of the tissue was done immediately after explantation at room temperature in the pathology department of the MHH. The dissected tissue was then cooled on ice and further kept at 4°C until its subsequent use for the determination of an *ex vivo* antibacterial activity.

Destruction of the tissue by homogenization processes, such as vortexing, was avoided. Instead, sampling of the mucopurulent secretion areas of the chronically *P*. *aeruginosa* infected CF lungs was achieved by collecting the secretions with the help of a sterile inoculation loop into 0.8 ml of PBS in a 2 ml eppendorf reaction tube. The weight of each of the collected mucopurulent secretion mass was recorded in order to adjust CFU determination relative to the weight of the patient´s material. Multiple experimental replicates were prepared for each of the tested treatment condition (PBS control, tobramycin 16 μg ml^-1^, PMZ 100 μM, and the combination of the two (PMZ and TOB)). The *ex vivo* mucus biofilms were incubated in the treatment solutions for 24 h at 37°C without shaking, and the material was then resuspended by vortexing the tubes for 30–60 sec. Serial dilutions of the resuspended biofilm were prepared in PBS and 100 μl of the dilutions were plated onto LB agar and selective *Pseudomonas* Cetrimide Agar. The plates were then incubated for at least 24 h at 37°C to obtain CFU/mg mucus.

### *In vivo* murine tumor model

Experiments were performed as described previously [50]. In brief, seven to eight-week old female BALB/c mice (Janvier, Germany) were injected intradermally into the left flank with 5x10^5^ of CT26 colon carcinoma cells or F1A11 fibro-sarcoma cells in 100 μl PBS. After roughly 10 days the tumors had grown to a volume of 150–200 mm^3^. The mice were then infected with 5x10^6^ *P*. *aeruginosa* PA14 intravenously (i.v.). Under these conditions, the bacteria colonize preferentially the tumors while spleen and liver will bear 100-to 1000-fold less bacteria. Systemic application of such bacteria induces rapid release of cytokines with TNF-α being most dominant. This results in a severe hemorrhage in the tumor and the formation of a large necrotic region in the center of the tumor. *P*. *aeruginosa* proliferate in this necrotic area but especially in the region between necrosis and the remaining viable, hypoxic interface. We could show that in this region the bacteria form biofilms [51,52]. Interestingly, from transcriptional profiling we conclude that for the bacteria this microenvironment very closely resembles the environment they encounter in the cystic fibrotic lung [50]. Experimentally, robust bacterial biofilms are established within the tumor by 48 h. Therefore, colonized tumor bearing mice were treated locally intra-tumorally (i.t.) with 50mg kg ^-1^ PMZ once or twice with 24 h intervals. Starting at the same time, tobramycin (2mg kg-^1^ in 100μl PBS) was given i.v. three or five times at 12 h intervals as specified. Six h after the last application, the mice were euthanized by CO_2_ asphyxiation and tumors isolated. The tumors were homogenized in 0.1% (v/v) Triton X-100/PBS using gentle MACS M-tubes and a dissociator from Miltenyi Biotec. The samples were serially diluted and plated on LB agar plates containing ampicillin (0.1 mg ml^-1^).

### Statistical analysis

All experiments were performed at least in biological triplicates, and with three technical replicates per experiment. Statistical analysis was made using GraphPad Prism version 5 (GraphPad Software, La Jolla, California, USA, www.graphpad.com).

## Results

### PMZ sensitizes biofilm-grown bacteria to antibiotic killing

**Fig 1:**
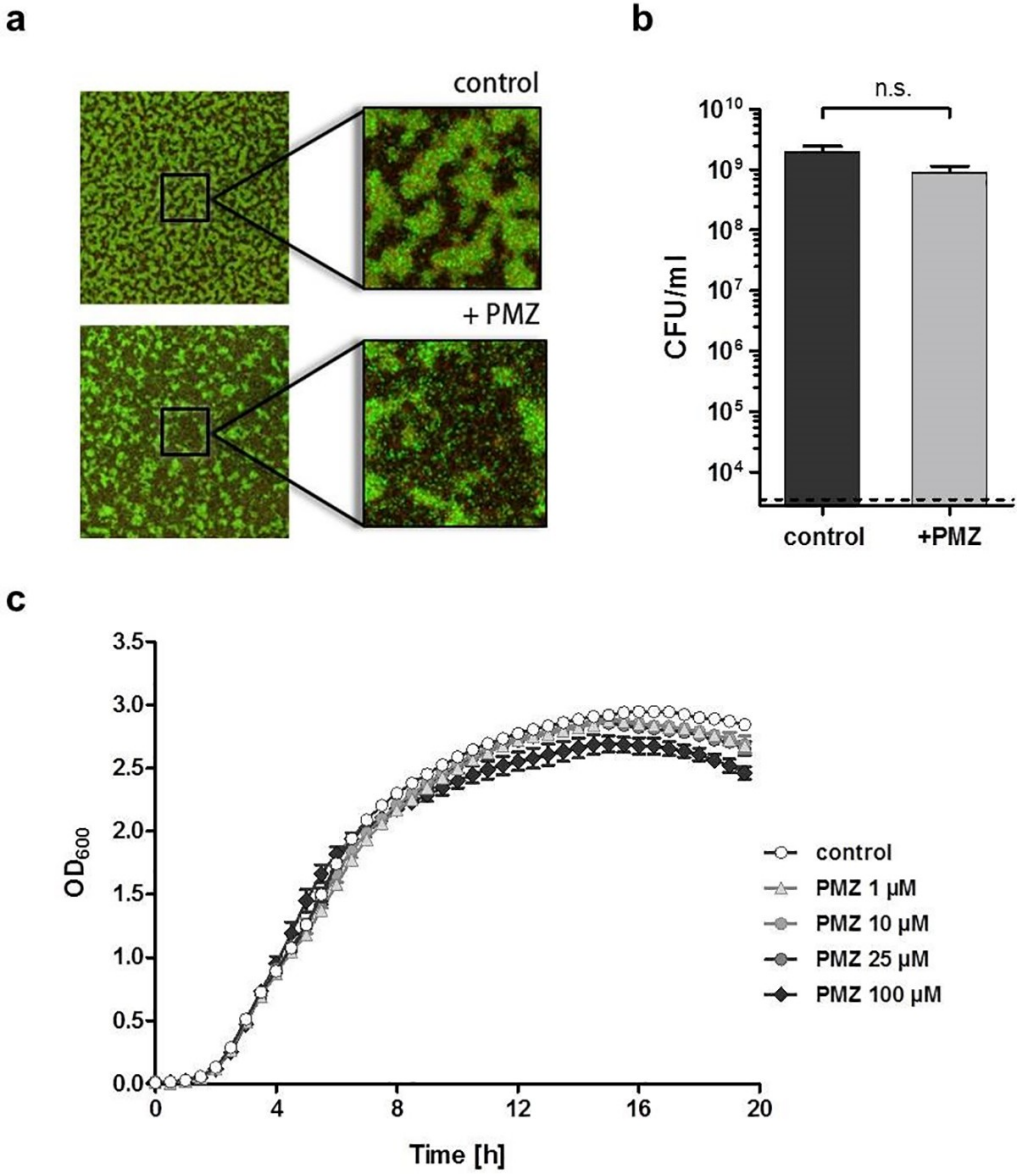
PMZ does not affect bacterial growth nor viability. 100 μM promethazine (PMZ) slightly influenced the structure, but not the viability of biofilm-grown *P*. *aeruginosa* cells. (**a**) Representative maximum intensity projections of PA14 biofilms treated with PMZ for 24 h in comparison to untreated control biofilms. The biofilms were stained with the *Bac*Light Bacterial Viability Kit, visualizing dead cells in red (propidium iodide) and living cells in green (Syto9). (**b**) Determination of colony forming units (CFU) from the untreated (dark grey) and PMZ-treated (light grey) biofilms revealed no significant differences in CFU/viability (Significance was calculated using the t- test). Data show mean CFU counts from overall 6 independent experiments. (**c**) Increasing concentrations of PMZ (1 μM, 10 μM, 25 μM and 100 μM) marginally affected planktonic growth of *P*. *aeruginosa* PA14 cells. Experiments were performed three times with at least three biological replicates each; here an exemplary data set from one experiment is shown.

In order to test the anti-biofilm activity of PMZ, the *P*. *aeruginosa* type strain PA14 was grown in 96-well plates for 24 h to establish biofilms prior to the addition of 100 μM PMZ with and without additional antibiotics. Following live/dead staining, the bactericidal activity of the compounds on biofilm-grown PA14 was monitored by acquiring confocal images at two positions in duplicate wells. Despite modest effects on the structure of *P*. *aeruginosa* biofilms, no profound PMZ-mediated effects on the viability of the biofilm bacteria were observed (Fig 1A and 1B). PMZ did not exhibit an antibiotic activity on planktonic bacteria, and growth was not altered at PMZ concentrations as high as 100 μM (Fig 1C).

**Fig 2:**
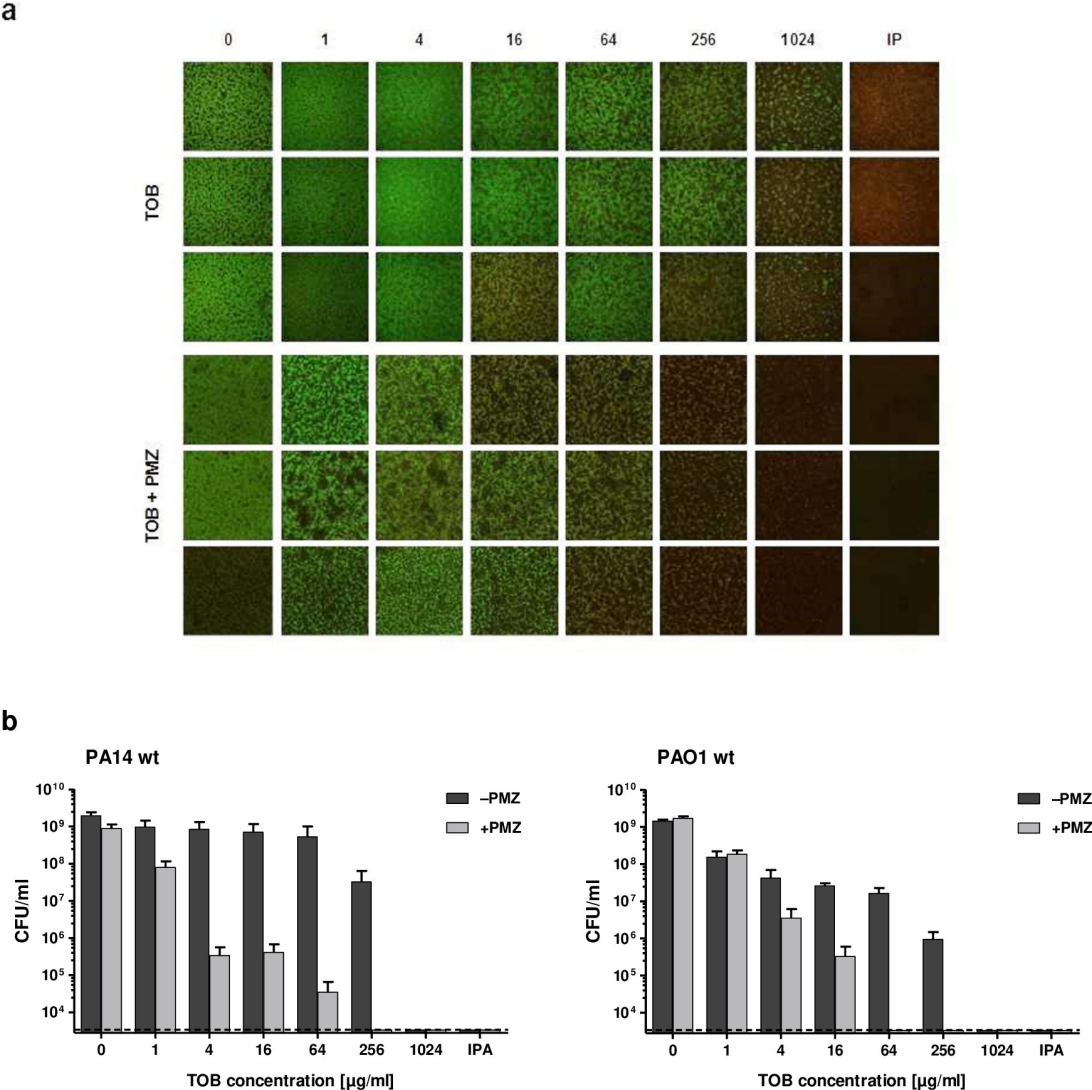
Synergistic anti-biofilm activity of PMZ in combination with tobramycin. *P*. *aeruginosa* static biofilms grown in 96-well plates were treated with increasing concentrations of tobramycin (TOB) with or without the addition of 100 μM promethazine (PMZ). (**a**) Representative maximum intensity projections of PA14 biofilms (triplicates) treated with TOB alone (upper panel), or in combination with PMZ (bottom panel). The biofilms were stained with the *Bac*Light Bacterial Viability Kit, visualizing dead cells in red (propidium iodide) and living cells in green (Syto9). Isopropanol treatment (IPA) was used as a killing control. Biofilms were assessed in triplicate wells, and at least three independent experiments were performed. (**b**) To assess the viability of the bacteria upon synergistic treatment, colony forming units (CFU) were determined from the tobramycin treated (dark grey) and tobramycin plus PMZ treated (light grey) biofilms of *P*. *aeruginosa* PA14 as well as the PAO1 reference strain. Mean CFU counts were performed on pools of three wells in at least three independent experiments. Error bars represent the standard error of the mean, while the dashed line indicates the lower limit of detection of the assay.

However, treatment of *P*. *aeruginosa* PA14 and PAO1 biofilms with PMZ, in combination with tobramycin, strongly enhanced the killing activity of tobramycin (Fig 2). Up to a four-log reduction of viable bacteria were observed in biofilms treated with a combination of PMZ and tobramycin, in comparison to tobramycin alone. The sensitizing activity of PMZ was not restricted to tobramycin, but was also observed for ciprofloxacin, ceftazidime and gentamicin (S1A, S1B and S1C Fig). Furthermore, we tested the sensitizing effect of PMZ on *Escherichia coli K12* cultures. We observed a synergistic, bactericidal effect when *E*. *coli* biofilm-grown cells were treated with PMZ and tobramycin; albeit, the effect was less pronounced than in *P*. *aeruginosa* cultures (S1D Fig).

### PMZ sensitized biofilm-grown cells can be efficiently killed at MIC concentrations

Since the combined use of antibiotics and PMZ killed the majority of *P*. *aeruginosa* biofilm-associated bacteria, when the antibiotics were added at MICs concentrations, we tested how an increase in the MIC would affect killing of biofilm-associated cells. We generated two ciprofloxacin resistant strains by introducing a target mutation into the *gyrA* gene individually (MIC of 2 μg/ml), and in combination with a target mutation in *parC* (MIC of 32 μg/ml). The introduction of these two target mutations drastically elevated the MIC of ciprofloxacin from that measured for WT cultures (0.125 μg/ml). Additionally, we used a PA14 transposon mutant that harbored a gentamicin resistance cassette, which increased the MIC of gentamicin from 2 μg/ml to >2048 μg/ml. The three resistant strains were grown under biofilm conditions and killing by the respective antibiotics was monitored in PMZ-sensitized cells. Both, ciprofloxacin and gentamicin, exhibited synergistic activities on biofilm-grown bacteria with PMZ, even in the resistant PA14 strains. For bacteria of such strains exhibiting increased MIC values, at least MIC concentrations were required to sufficiently kill the biofilm-grown PA14 variants (S2 Fig). In conclusion, PMZ reverts antimicrobial tolerance in all the strains tested under biofilm growth conditions. Thus, biofilm-grown cells can be effectively killed at MIC concentrations. Differences in antimicrobial killing of planktonic and biofilm-grown bacteria level off.

### PMZ–mediated synergistic activity is enhanced in PQS producing cells

We observed that clinical *P*. *aeruginosa* isolates and mutant strains that exhibited low or abolished expression of the *Pseudomonas* quinolone signaling (PQS) system were less prone to sensitization towards antimicrobial killing upon PMZ treatment. Interestingly, it has previously been demonstrated that increases in the levels of PQS, or decreases in the ability to induce an oxidative stress response, influence the antibiotic susceptibility of biofilm-grown *P*. *aeruginosa* isolates [53]. Here, the treatment of biofilms of a *pqsA* negative mutant, which is not able to produce PQS signaling molecules, revealed that there is significantly diminished sensitizing activity of PMZ towards the bactericidal activity of tobramycin (S3 Fig; compare to results of Fig 2B). In line with this, we observed that *E*. *coli* biofilms were sensitized to a lower extent by PMZ towards tobramycin killing activity (S1 Fig). The exogenous addition of PQS to the non-producing *P*. *aeruginosa* mutant restored the sensitizing activity of PMZ in *pqsA* mutant biofilms (S3 Fig). Furthermore, also in *E*. *coli* biofilms more cells were killed by PMZ/tobramycin if the biofilms were simultaneously treated with PQS. Thus, it seems that strong synergistic PMZ/tobramycin anti-biofilm activity in *P*. *aeruginosa* is dependent on the presence of PQS. Since PQS enhanced PMZ/tobramycin killing not only in *P*. *aeruginosa* but also in *E*. *coli* biofilm cells, this implicates that the activity of PQS per se and not its impact on bacterial signaling contributes to the killing activity.

### Synergistic activity of PMZ is dependent on the pH of the medium

**Fig 3:**
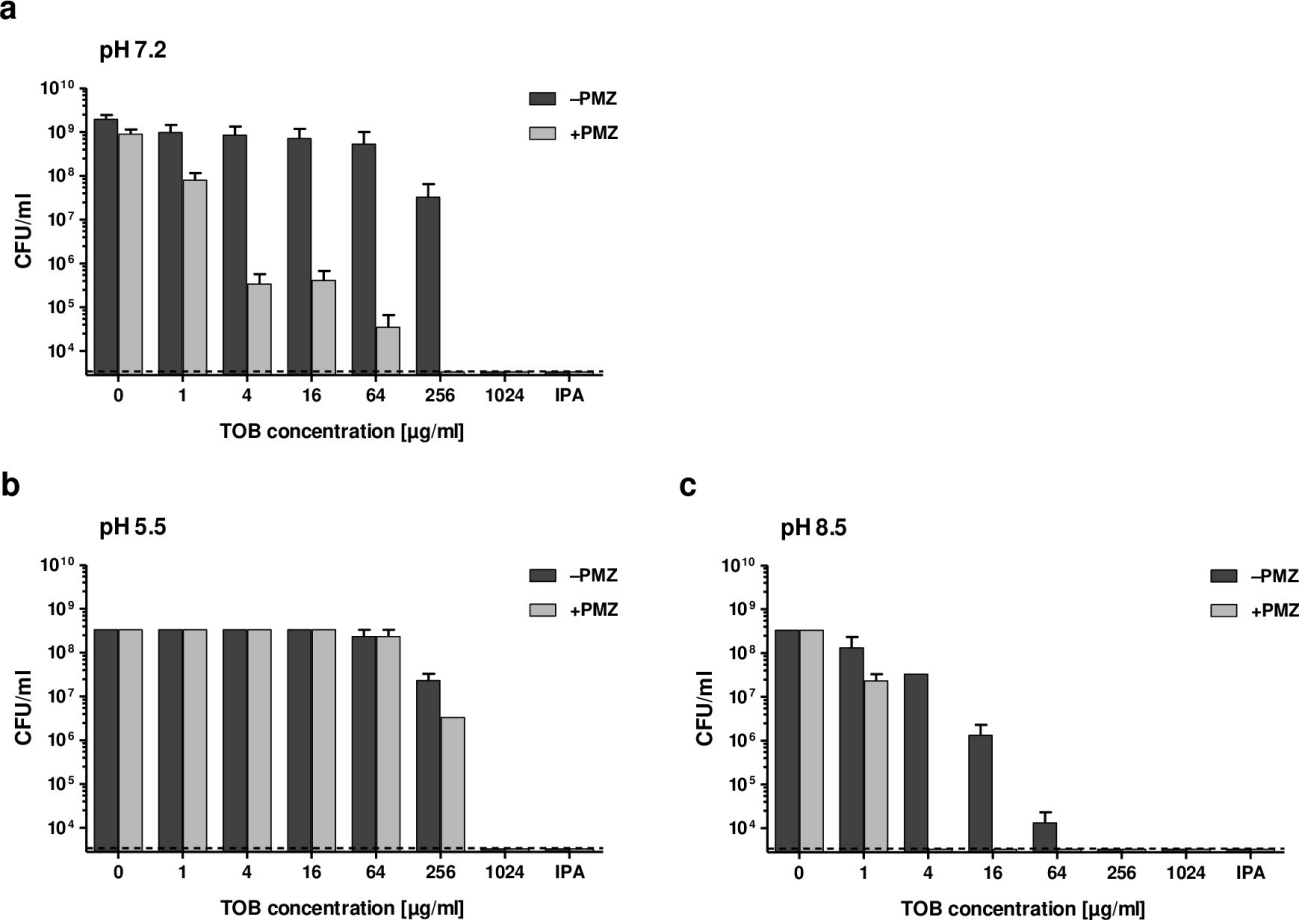
The pH of the growth medium affects the synergistic anti-biofilm activity of PMZ in combination with tobramycin. *P*. *aeruginosa* static biofilms grown in 96-well plates were treated with increasing concentrations of tobramycin (TOB) with or without the addition of 100 μM promethazine (PMZ) in buffered LB. The pH of the medium was buffered to 7.2 (**a**) 5.5 (**b**) or 8.5 (**c**), respectively. Colony forming units (CFU) were determined from the tobramycin treated (dark grey) and tobramycin plus PMZ treated (light grey) biofilms. Isopropanol treatment (IPA) was used as a killing control. Mean CFU counts were performed on pools of three wells in at least three independent experiments. Error bars represent the standard error of the mean, while the dashed line indicates the lower limit of detection of the assay.

The extracellular pH has previously been demonstrated to have an impact on the activity of various antibiotics [54,55]. Therefore, we repeated the biofilm killing experiments with PA14 biofilms grown in pH buffered medium to stabilize the pH at 5.5, 7.2, and 8.5, respectively. An increase or decrease in the pH of the medium strongly influenced the susceptibility of PA14 biofilms to the synergistic effects of PMZ and tobramycin. In accordance to previous studies, the killing activity of tobramycin was more effective under higher pH medium conditions [54,55]. At a pH of 8.5, the combination of PMZ with tobramycin reduced the CFU counts below the detection limit at tobramycin concentration levels as low as 4 μg/ml, whereas 256 μg/ml was needed at pH of 7.2 and even 1024 μg/ml at pH of 5.5 (Fig 3).

### PMZ increases the membrane potential and results in a decreased oxygen consumption and extracellular acidification rate of *P*. *aeruginosa* cells

**Fig 4:**
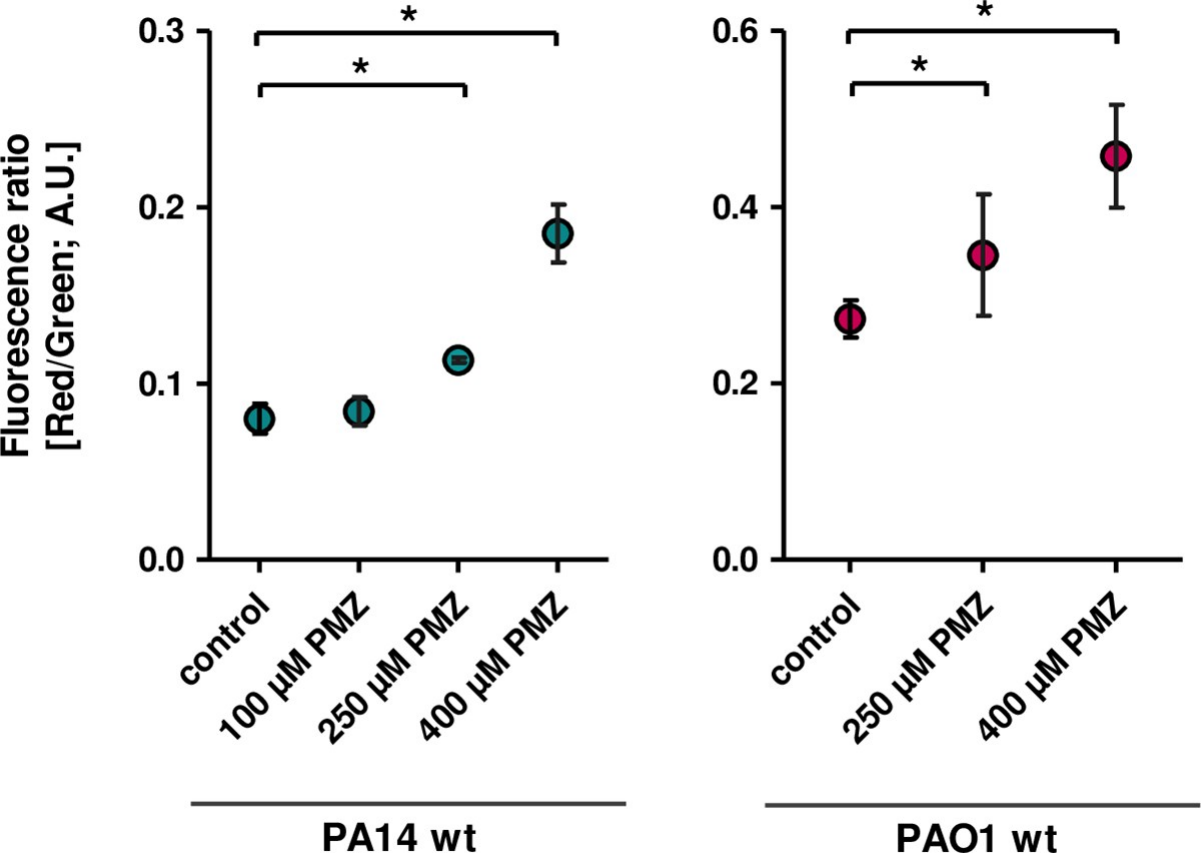
PMZ increases the membrane potential of *P*. *aeruginosa*. Exponentially growing cells of PA14 (left) and PAO1 (right) were challenged with increasing concentrations of PMZ. Cells were stained with the membrane potential indicator DiOC_2_(3) and analyzed by flow cytometry. The red/green fluorescence ratio was calculated using population mean intensities. Experiments were carried out in triplicates. Data analysis by Student’s t-test demonstrates significant difference (* *p* < 0.05) between the treated samples and the control.

PMZ is known to interfere with the transport of ions across the membrane [56,57]. Thus, we measured the membrane potential of *P*. *aeruginosa* cells (PA14 and PAO1) following the addition of PMZ. An increase in the membrane potential in a concentration dependent manner was observed (Fig 4).

**Fig 5:**
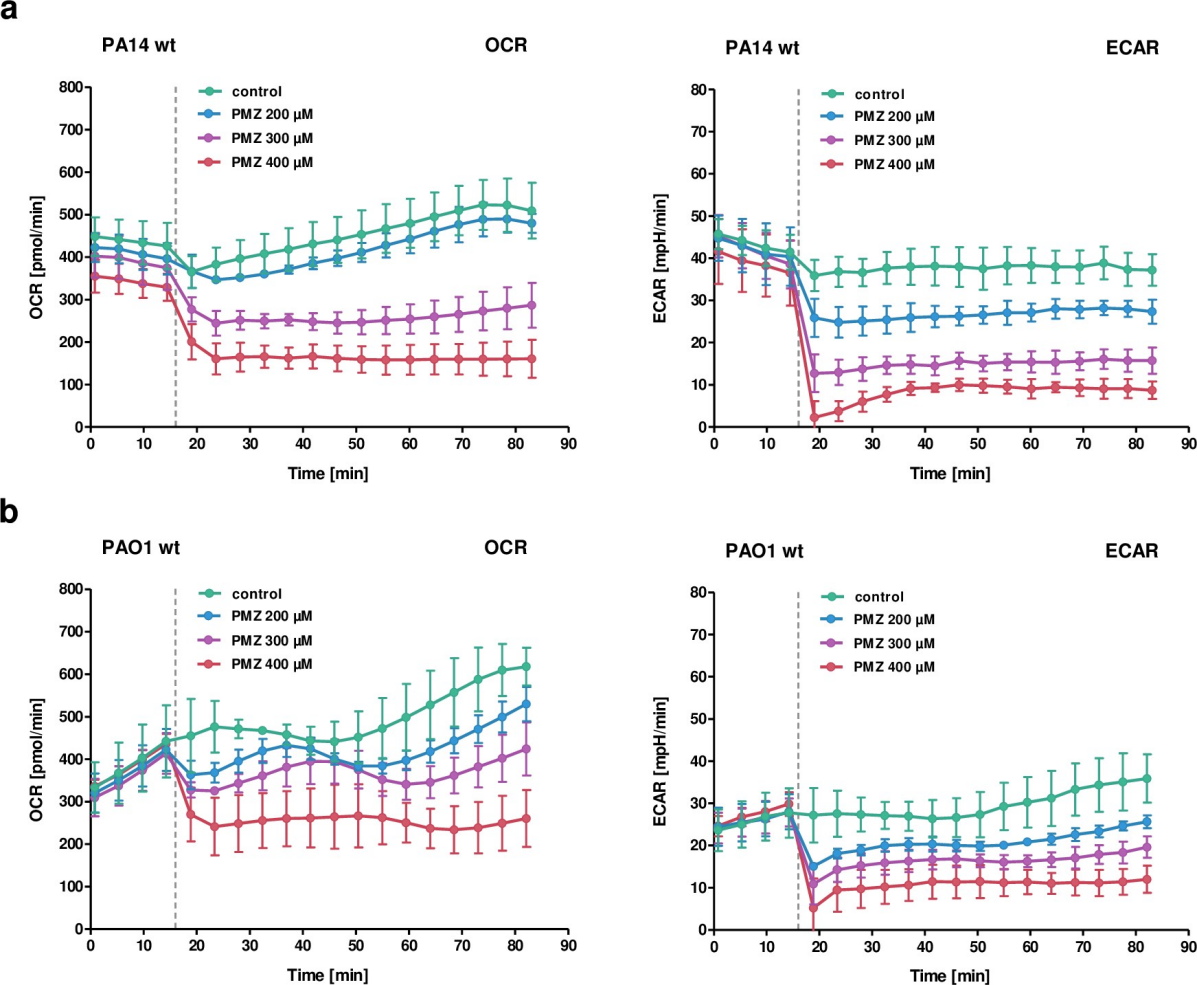
Bioenergetic analysis of PA14 upon addition of PMZ. Oxygen consumption rate (OCR; left) and extracellular acidification rate (ECAR; right) of *P*. *aeruginosa* PA14 (a) and PAO1 (b) treated with increasing concentrations of PMZ. The dotted line indicates the addition of PMZ after four cycles of basal OCR and ECAR measurements (16 minutes). Data show the mean and standard deviation of three technical replicates, calculated by the Seahorse XF Wave software. One representative experiment is shown of at least three independent experiments. The data were corrected for background artifacts from sole addition of PMZ to cell-free buffer medium (see Experimental Procedure).

Membrane hyperpolarization can create “backpressure” on the proton pumping complexes of the electron transport chain, thereby slowing their function [58]. To measure the oxygen consumption rate (OCR) and the extracellular acidification rate (ECAR) of *P*. *aeruginosa* treated with increasing concentrations of PMZ, we used the *Seahorse* XFe96 Analyzer (Agilent). Immediately following PMZ exposure, both the OCR and the ECAR dropped in a concentration dependent manner Fig 5.

### CCCP reduces the synergistic killing activity of PMZ in combination with tobramycin, whereas extracellular potassium addition increases it

Changes in extracellular pH, and the addition of PMZ, affect the electrochemical gradient across the membrane. Therefore, we wondered whether *P*. *aeruginosa* becomes sensitive to bactericidal antibiotics upon an increase in membrane potential. To test this, we repeated the biofilm killing experiments in medium supplemented with 300mM KCl as well as the uncoupler/ionophore, CCCP (carbonyl cyanide-m-chlorophenyhydrazon), both of which decrease the membrane potential.

**Fig 6:**
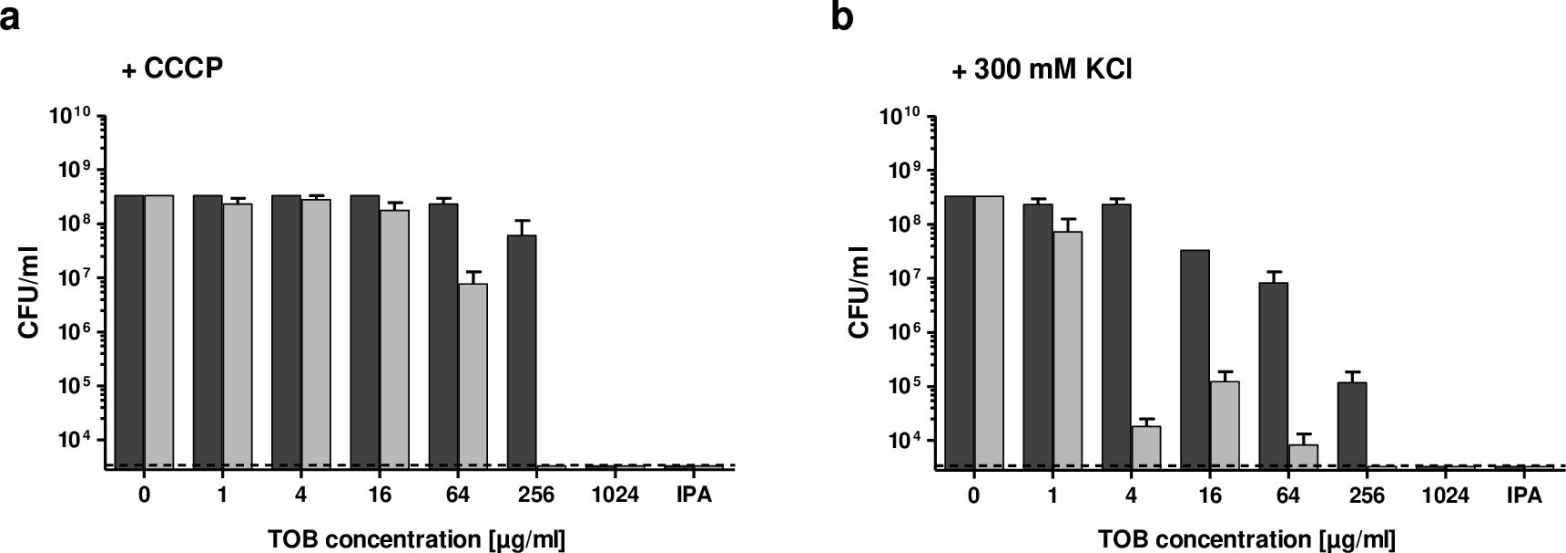
High extracellular potassium and the addition of CCCP affect the synergistic anti-biofilm activity of PMZ in combination with tobramycin. *P*. *aeruginosa* PA14 biofilms were treated with increasing concentrations of tobramycin (TOB) alone or in combination with 100 μM PMZ and further challenged with the protonophore CCCP (**a**), or with high extracellular potassium (300 mM KCl) (**b**). Colony forming units (CFU) were determined from the tobramycin treated (dark grey) and tobramycin plus PMZ treated (light grey) biofilms. Isopropanol treatment (IPA) was used as a killing control. Mean CFU counts were performed on pools of three wells in at least three independent experiments. Error bars represent the standard error of the mean, while the dashed line indicates the lower limit of detection of the assay.

**Fig 7:**
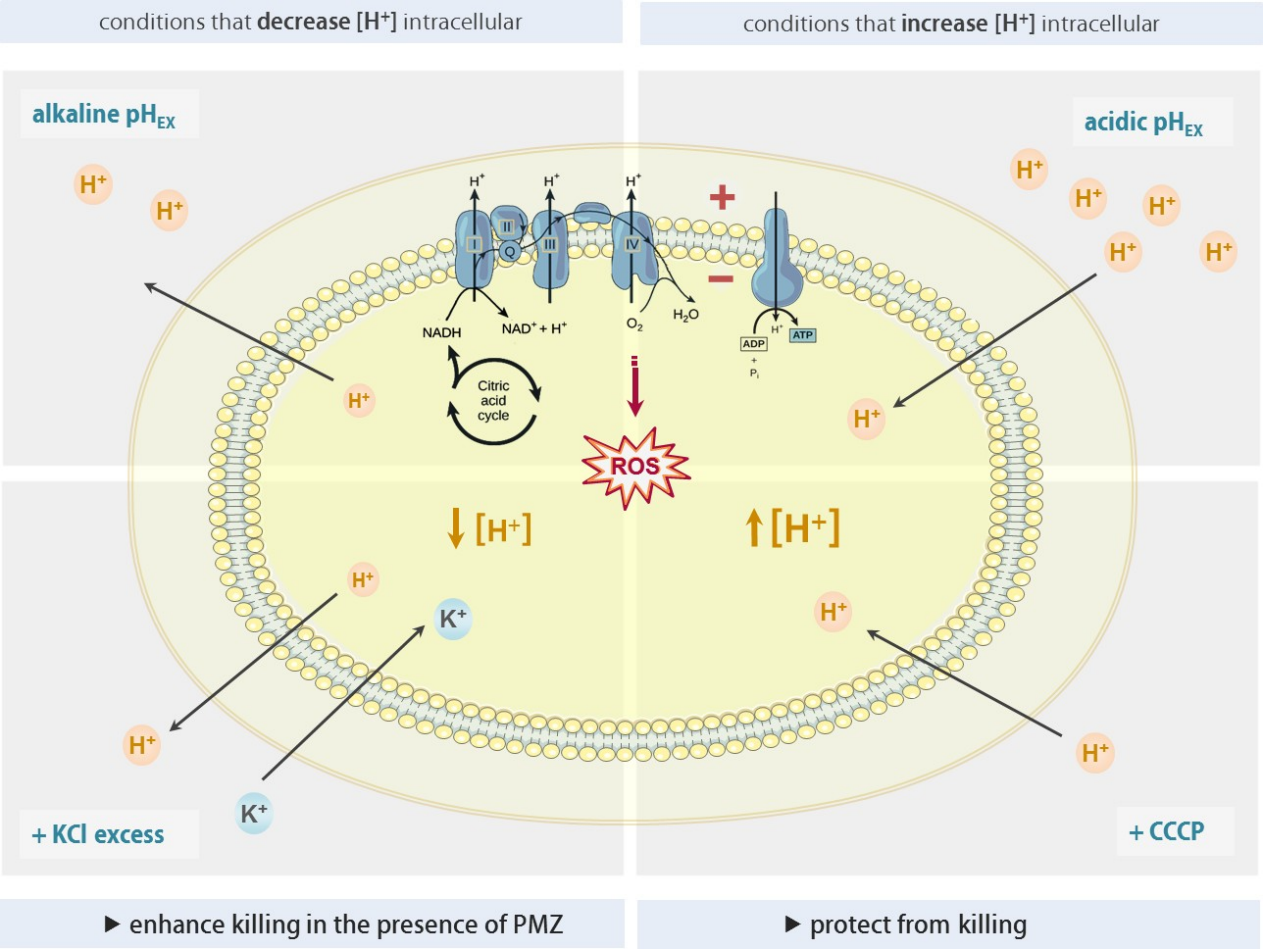
The levels of intracellular protons modulate the synergistic killing activity of PMZ and antibiotics.

The addition of CCCP reduced the synergistic killing activity of PMZ in combination with tobramycin. However, high extracellular potassium concentrations increased the synergistic activity of tobramycin and PMZ (Fig 6). Interestingly, CCCP increases the membrane permeability for protons, leading to a net flow of protons into the cell; whereas under high extracellular potassium concentrations, the flow of protons is reversed, and there is a net flow of protons out of the cell. Our results indicate that a change in proton concentration, rather than a change in membrane potential, enhances the synergistic activity of PMZ and antibiotics on biofilm-associated bacteria. Decreasing the intracellular levels of protons by increasing the extracellular pH, or by increasing the extracellular potassium concentrations, increases the bactericidal effect of antibiotics. In contrast, a decrease in the extracellular pH, or an increase in membrane permeability, increases intracellular proton levels, thereby reducing the efficacy of antibiotics (Fig 7).

### Tobramycin and PMZ combination therapy reduces bacterial burden in an in vivo biofilm model

**Fig 8:**
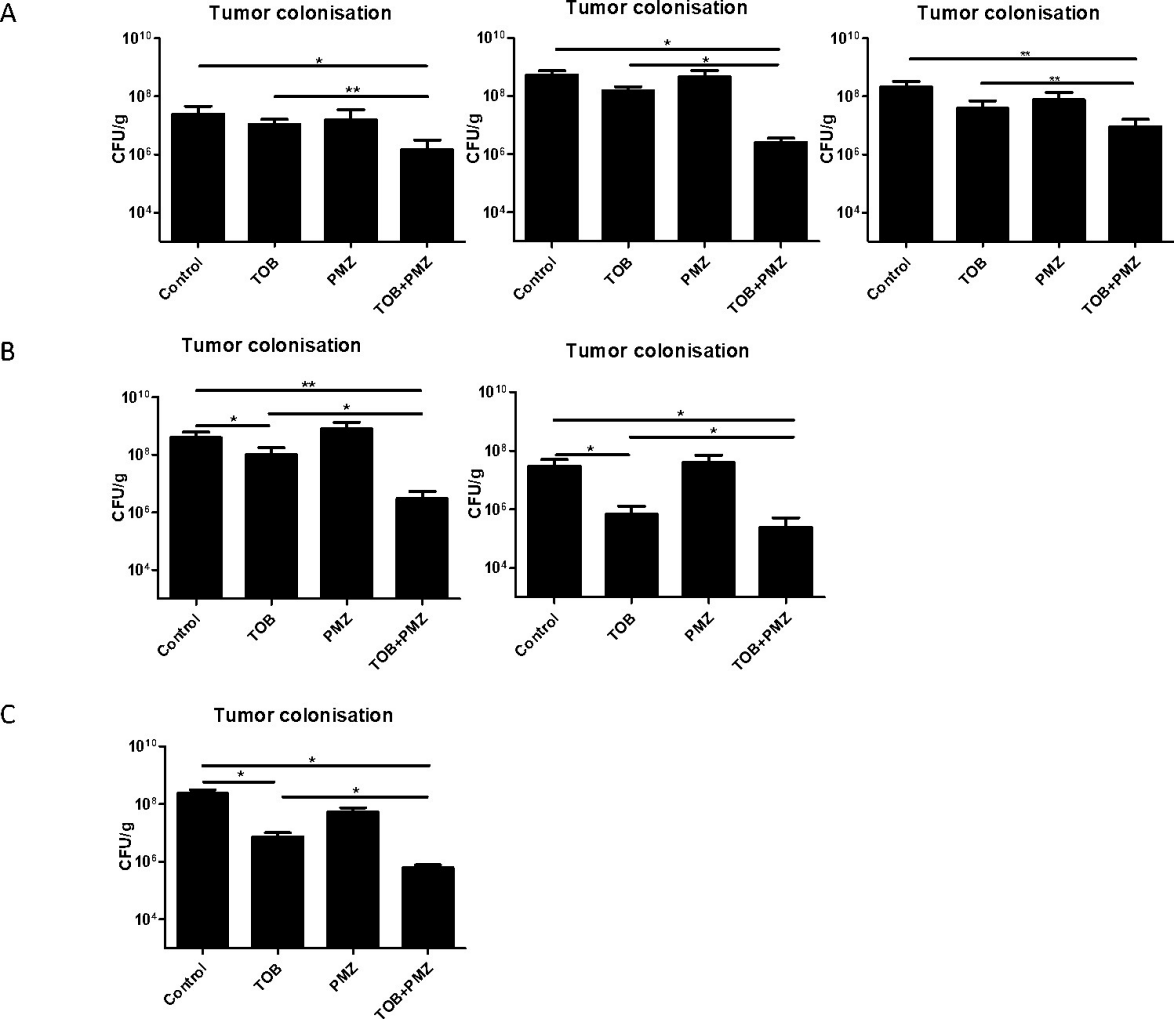
*In vivo* synergistic activity of tobramycin and PMZ on biofilm forming *P*. *aeruginosa*. (A) PA14 colonized CT26 tumor bearing mice were treated with 50 mg kg^-1^ PMZ by single i.t. injection followed by three i.v. injections of 2mg kg^-1^ tobramycin at 12 h intervals. (B) PA14 colonized CT26 tumor bearing mice were treated twice with 50 mg kg^-1^ PMZ at an interval of 24 h via i.t. injections. Starting at the same time, five i.v. injections of 2mg kg^-1^ tobramycin at 12 h intervals were applied. (C) PA14 colonized F1A11 tumor bearing mice were treated twice with 50 mg kg^-1^ PMZ at an interval of 24 h via i.t. injections combined with five i.v. injections of 2mg kg^-1^ tobramycin at 12 h intervals. The panels represent individual experiments. At least 5 mice per group were tested. Tumors were harvested 6 h after final administration, homogenized and plated for CFU quantitation. *, P < 0.05; **, P < 0.005 (P value were determined by unpaired t test).

We next evaluated the synergistic activity of PMZ and tobramycin on the killing of biofilm-associated *P*. *aeruginosa* in an *in vivo* system. We therefore used our previously established murine tumor model for biofilm infections. According to the genetic profile of the bacteria, the tumor model very closely simulates the environment of the cystic fibrosis lung for the bacteria (50). Mice bearing CT26 tumors were infected i.v. with *P*. *aeruginosa*. This leads to stable biofilm formation within two days post infection within the tumor. The mice were then treated with a combination of PMZ and tobramycin. In the first experiment, mice bearing CT26 tumors colonized with biofilm-residing *P*. *aeruginosa* were treated once i.t. with PMZ and three times i.v. with tobramycin at 12 h intervals. Mice were sacrificed and CFU numbers in the tumors and the liver were determined by plating tissue homogenates. A reduction of bacterial load was observed at 2 mg kg^-1^ tobramycin in the tumor (Fig 8A). The reduction in CFU counts was drastically more pronounced in the tumor of mice that had been treated with tobramycin in combination with PMZ.

In an attempt to optimize the application regimen, i.t. application of PMZ was performed twice within a 24 h interval with mice bearing colonized CT26 tumors. Tobramycin was applied five times i.v. with 12 h hour intervals. Under these circumstances already an antibiotic effect could be observed (Fig 8B) as described before (51). Nevertheless, an enhanced effect of the combination therapy could still be observed (Fig 8B). To test this therapy in a tumor system less prone to bacterial toxicity, we switched to the aggressive fibrosarcoma F1A11 keeping the other experimental condition constant. Again, an effect of the antibiotics alone could be observed (Fig 8C). However, the enhanced effect of the combination of PMZ and tobramycin became much more obvious (Fig 8C). Thus, as predicted from the *in vitro* data when tobramycin was administered in combination with PMZ, the efficacy of tobramycin against biofilm residing bacteria in the tumor model can be strongly enhanced when combined with PMZ.

### Treatment of *P*. *aeruginosa* microcolonies from chronic CF lung infections with PMZ/tobramycin

**Fig 9:**
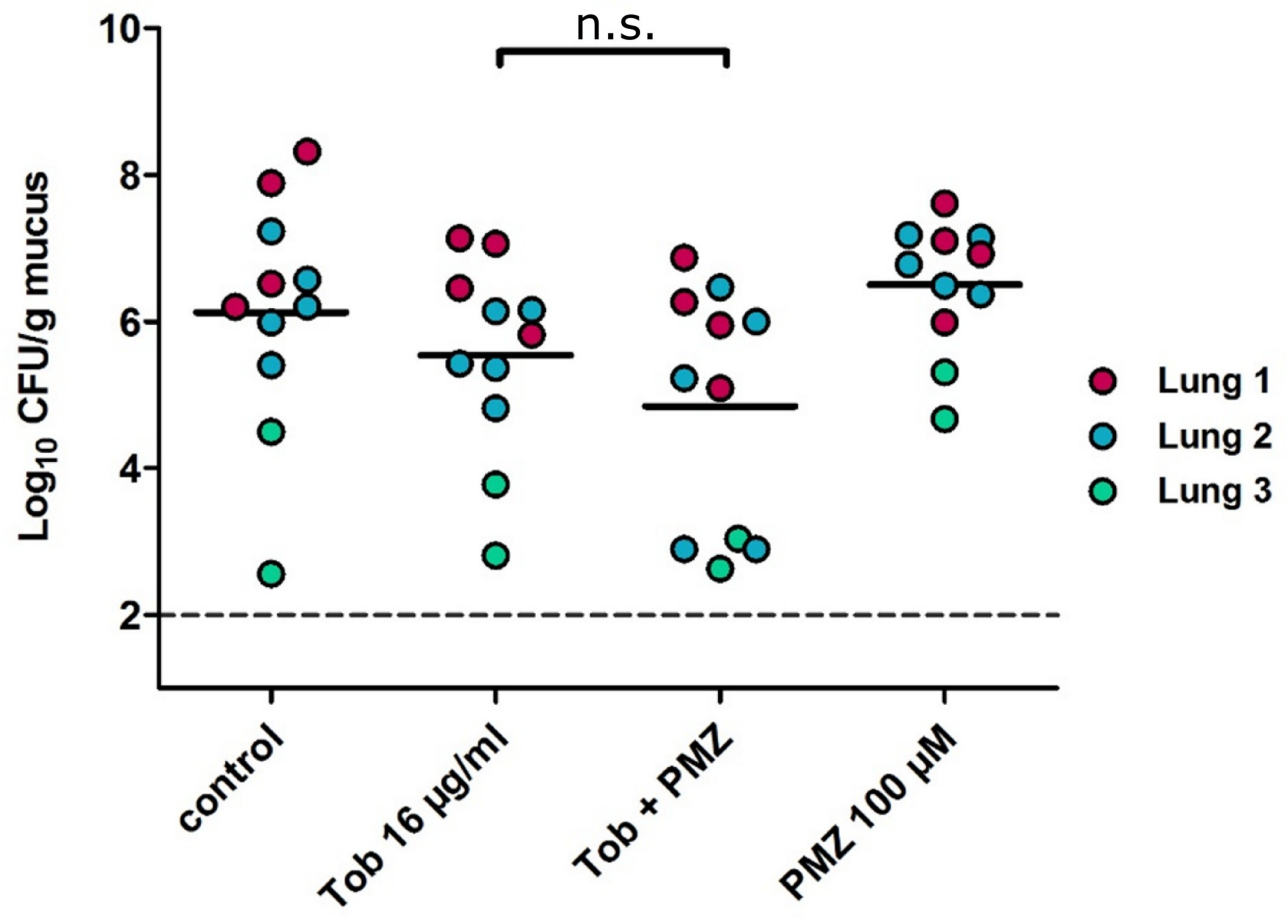
PMZ and tobramycin show a synergistic killing in *P*. *aeruginosa* infected *ex vivo* lung tissue samples. Tissue samples were collected from three explanted CF lungs which were found to be chronically infected by *P*. *aeruginosa*. Tobramycin was added to the infected tissue at a final concentration of 16 μg/ml with and without 100 μM PMZ. Colony forming units (CFU) were determined in the PMZ, tobramycin and tobramycin plus PMZ treated samples. Each colored dot represents a different technical replicate of tissue samples (coming from the same lung) whereas the different colors are biological replicates (different lungs). The dashed line indicates the lower limit of detection of the assay. Horizontal bars represent the mean. No significant differences were observed between the tobramycin treated samples and those treated with tobramycin in combination with PMZ (Mann Whitney U test).

To further evaluate the synergistic activity of PMZ and tobramycin on the killing of biofilm-associated *P*. *aeruginosa*, we tested their bactericidal activity on human *ex vivo* samples. We collected tissue samples from three chronically infected explanted cystic fibrosis lungs and exposed the samples to tobramycin, or tobramycin in combination with PMZ. Despite a trend, the combined use of PMZ and tobramycin did not show a significant synergistic bactericidal activity on the *ex vivo P*. *aeruginosa* biofilm samples (Fig 9). More work needs to be done, in order to evaluate the optimal synergistic concentrations of PMZ and tobramycin in an *in vivo/ex vivo* setting, to improve delivery of the drug and to adjust environmental conditions (such as pH and efficient delivery of the drug) to promote the synergistic killing activity.

## Discussion

The general recalcitrance of biofilm-associated bacteria diminishes the efficacy of antimicrobials. Accordingly, present antimicrobial treatment protocols that serve well for the eradication of acute infections fail to clear biofilm-associated chronic infections. As a result, morbidity and mortality due to chronic infections have remained unchanged over the past few decades. Alternative therapeutic strategies to eradicate biofilm-associated infections are desperately needed.

Many efforts to decrease the burden of chronic infections have been made; however, despite the initial enthusiasm and the vast amount of literature on the identification of novel anti-biofilm compounds, no antimicrobials are currently in clinical use to specifically treat biofilm-associated infections. An alternative way to meet the clinical need could be to concentrate on enhancing the activity of our current antibiotics [35,59]. It seems that the successful eradication of biofilm-associated bacterial infections relies on our ability to break the antibiotic tolerance of the biofilm-grown bacteria.

In this study, we show that PMZ targets antimicrobial tolerance under biofilm growth conditions. We found that in the opportunistic pathogen *P*. *aeruginosa*, PMZ fully restores the bactericidal activity of common antibiotics in *in vitro* grown biofilm bacteria and to some extent *in vivo*. The finding that PMZ breaks tolerance against several classes of antibiotics seems to be consistent with a shared underlying molecular tolerance mechanism.

It is well established that hypoxia [60], low nutrient availability [53,61], and low pH [55,62] have the potential to shift a pathogen into a drug-tolerant state [63]. The effectiveness of antibiotics appears to depend on bacterial growth and metabolism and there is considerable evidence that dysregulation of bacterial bioenergetics is important in modulating drug lethality [64–66]. Extensive screens for genes that contribute to antibiotic resistance in *P*. *aeruginosa* [67, 68] and other bacterial pathogens [69,70] have revealed that mutations influencing the production of NADH (by e.g. interrupting TCA cycle genes) or the subsequent NADH oxidation in the electron transport chain are responsible for a tolerant phenotype. Previous work also identified a link between antibiotic-induced cellular respiration and bactericidal lethality. Bactericidal activity can be arrested by attenuated respiration and potentiated by accelerated respiration [71], and has been proposed to be connected to enhanced oxidative stress generated from increased aerobic respiration [47,72]. Accordingly, killing of biofilm-associated bacteria can be counteracted by molecular mechanisms that reduce oxidative stress within the cells [53].

Recently, it was also proposed that in antibiotic-treated bacterial cells in the absence of reduced respiration, protons would be drained from the cytoplasm and not replaced through proton re-entry via ATP synthesis as *the cells* switch from high to *low ATP* states [54,58]. It was suggested that the effects of *antibiotics* on *pH homeostasis* should be considered a potential mechanism that contributes to *antibiotic* lethality. However, as the chemistries of protons and electrons are closely linked, it may be not possible to decipher if the bactericidal effect of an antibiotic is due to oxidative stress, or the disruption of pH homeostasis.

In accordance with the pH homeostatic model, we demonstrate that the antibiotic tolerance of biofilm-grown cells can be reversed under conditions that induce low concentrations of intracellular protons by increasing the extracellular pH, or extracellular potassium concentrations. Antibiotic activity was counteracted by increasing the intracellular levels of protons via decreasing the extracellular pH or increasing the membrane permeability for protons (addition of CCCP). Thus, changes in environmental conditions, which lead to decreased intracellular proton levels, promoted antibiotic killing of biofilm-grown bacteria. Of note, this killing could be clearly enforced by the addition of PMZ. We demonstrate that the simultaneous treatment of *in vitro* grown biofilm bacteria with PMZ and tobramycin was sufficient to fully revert the biofilm-growth mediated tolerance phenotype and tobramycin killed biofilm-grown cells at MIC concentrations.

This is remarkable because the PMZ-mediated inhibitory effect on cellular respiration curtails cellular alkalization. However, PMZ induced membrane hyperpolarization also leads to conditions that are energetically less favorable for proton-pumping electron transport chain complexes. While this hyperpolarization counteracts alkalization of the cytoplasm, it may also increase the formation of ROS due to the stalling of electrons on the electron transport chain complexes. Our finding that the activity of PMZ sensitization was diminished in *P*. *aeruginosa* strains with low or abolished production of PQS supports the finding that ROS production contributes to effective antibiotic killing. PQS is an iron scavenger and has been demonstrated to have anti- and pro-oxidant activities [73]. Its presence was determined to be essential for antibiotic killing of starved biofilm-grown cells [53].

It is also conceivable that the PQS governed 4-quinolone derivate 2-heptyl-4-hydroxyquinoline n-oxide (HQNO) contributes to the sensitization of the biofilm cells to the synergistic activity of PMZ and tobramycin. HQNO has previously been shown to be a cytochrome bc1 complex inhibitor and to promote stationary phase killing of *P*. *aeruginosa* populations due to an inhibition of the electron transfer chain leading to enhanced ROS production [74]. Of note, an interference of phenothiazines with the respiratory chain of *Mycobacterium tuberculosis* has been demonstrated previously [75].

In addition, the phenothiazines were shown to inhibit the activity of multi-drug efflux pumps and to disrupt biofilms [34,76–79]. Multi-drug efflux pumps play an essential role in adapted antibiotic resistance [80–82] and are specifically upregulated under biofilm growth conditions [83–86]. Thus, an accumulation of antibiotics in PMZ treated bacterial cells could contribute to the synergistic activity observed between PMZ and tobramycin.

In conclusion, our data suggest that reduced proton efflux in cells that exhibit decreased respiratory activity (due to their growth in a biofilm) contributes to general antimicrobial tolerance. This tolerance phenotype can be overcome if cells are subjected to environmental conditions that lead to lower intracellular proton levels. Under these conditions, PMZ acts synergistically with antibiotics to fully revert the tolerance phenotype of biofilm-associated bacteria by inducing hyperpolarization of the cellular membrane. In this study, the simultaneous addition of PMZ and tobramycin was not sufficient to eradicate the bacteria from the *in vivo* biofilms in mouse tumor tissue or within microcolonies that were recovered from chronically infected explanted CF lungs. Clearly, more work is needed to evaluate whether and how the *in vivo* environment could be influenced to promote low intracellular proton levels in biofilm-associated cells to maximize the synergistic activity of PMZ and antibiotics.

Our findings open new avenues, and hold promise that targeting tolerance mechanisms of biofilm-associated bacteria could become a practicable way to change the way physicians treat biofilm-associated infections. Furthermore, since PMZ is already used in the clinic, bioavailability and safety profiles for this drug are already available. Proven formulation and manufacturing routes and reasonably characterized pharmacology for this drug would also aid in repurposing this drug, thereby enabling the entry into clinical phases more rapidly, and at a lower cost than novel compounds. Repurposing of PMZ as an antibiotic sensitizer of biofilm-grown bacterial cells may foster its fast introduction into clinical practice to enhance the activity of existing antibiotics so that chronic biofilm-associated infections can be targeted more effectively.

## Supporting information

S1 Table *Pseudomonas aeruginosa* strains used in this study. (DOCX)

S1 Fig Synergistic anti-biofilm activities of PMZ in combination with different antibiotics. *P*. *aeruginosa* static biofilms grown in 96-well plates were treated with increasing concentrations of ciprofloxacin (CIP) (**a**), ceftazidime (CAZ) (**b**) or gentamicin (GEN) (**c**) with or without the addition of 100 μM promethazine (PMZ). Colony forming units (CFU) were determined from the antibiotics treated (dark grey) and antibiotics plus PMZ treated (light grey) biofilms. Isopropanol treatment (IPA) was used as killing control. Mean CFU counts were performed on pools of three wells in at least three independent experiments. Error bars represent the standard error of the mean, while the dashed line indicates the lower limit of detection of the assay. (**d**) Biofilms of *Escherichia coli* K12 were treated in the same manner with increasing concentrations of either tobramycin alone or in combination with promethazine, and CFU were determined accordingly.
(TIF)

S2 Fig Synergistic activities of PMZ in combination with antibiotics on biofilms of *P. aeruginosa* PA14 exhibiting increased MIC values against ciprofloxacin and gentamicin. *P*. *aeruginosa* PA14 isogenic mutants, which exhibited an increase in the minimal inhibitory concentration (MIC indicated by the arrow) against ciprofloxacin due to a target mutation in *gyrA* (**a**), *gyrA* and *parC* (**b**) and gentamicin due to a gentamicin harboring resistance cassette transposon insertion (**c**), were grown in 96-well plates and treated with increasing concentrations of ciprofloxacin (a, b) and gentamicin (c) with or without the addition of 100 μM promethazine (PMZ). Colony forming units (CFU) were determined from the antibiotics treated (dark grey) and antibiotics plus PMZ treated (light grey) biofilms. Isopropanol treatment (IPA) was used as a killing control. Mean CFU counts were performed on pools of three wells in at least three independent experiments. Error bars represent the standard error of the mean, while the dashed line indicates the lower limit of detection of the assay.
(TIF)

S3 Fig Diminished synergistic activity of PMZ in combination with tobramycin on biofilms of a PQS non-producing strains. **(a)** A *P*. *aeruginosa* PA14 *pqsA* transposon mutant was grown in 96-well plates and treated with increasing concentrations of tobramycin with or without the addition of 100 μM promethazine (PMZ). Colony forming units (CFU) were determined from the tobramycin treated (dark grey) and tobramycin plus PMZ treated (light grey) biofilms. Isopropanol treatment (IPA) was used as a killing control. External addition of PQS (100 μM) restored the killing activity of tobramycin/PMZ in the *P*. *aeruginosa* PA14 *pqsA* transposon mutant (**b**) and enhanced the killing activity of tobramycin/PMZ in *E*. *coli* K12 (**c**). Mean CFU counts were performed on pools of three wells in at least three independent experiments. Error bars represent the standard error of the mean, while the dashed line indicates the lower limit of detection of the assay.
(TIF)
