# Supplementary material for: Targeting bioenergetics is key to counteracting the drug-tolerant state of biofilm-grown bacteria
Source: PLoS Pathog. 2020 Dec 22;16(12):e1009126. doi: 10.1371/journal.ppat.1009126 (PMC7787680; doi:10.1371/journal.ppat.1009126)
Supplement: S1 Table — (DOCX) [file ppat.1009126.s001.docx]

Supplementary Table S1: *Pseudomonas aeruginosa* strains used in this study

| **Strain** | **Description** | **Reference** |
| --- | --- | --- |
| PA14 | wild type reference strain | Liberati *et al*., 2006 |
| PAO1 | wild type reference strain |  |
| PA14 *gyrA* T831 | SNP mutation at position T831 in the quinolone resistance-determining region (QRDR) of the gene *gyrA* (encoding DNA gyrase) | Bruchmann *et al*., 2013 |
| PA14 *gyrA* T831 *parC* S87L | SNP mutation at position T831 in the QRDR of the gene *gyrA* (encoding DNA gyrase); SNP mutation at position S87L in the QRDR of the gene *parC* (encoding topoisomerase IV) | Bruchmann *et al*., 2013 |
| PA14 *ladS*∷tn | *ladS* transposon mutant from the NR PA14 transposon mutant library; ID 38371; Gm^R^ | Liberati *et al*., 2006 |
| PA14 *pqsA*∷tn | *pqsA* transposon mutant from the NR PA14 transposon mutant library; ID 23621; Gm^R^ | Liberati *et al*., 2006 |

Gm, gentamycin resistant
