# Supplementary figures and images for: Targeting bioenergetics is key to counteracting the drug-tolerant state of biofilm-grown bacteria
Source: PLoS Pathog. 2020 Dec 22;16(12):e1009126. doi: 10.1371/journal.ppat.1009126 (PMC7787680; doi:10.1371/journal.ppat.1009126)

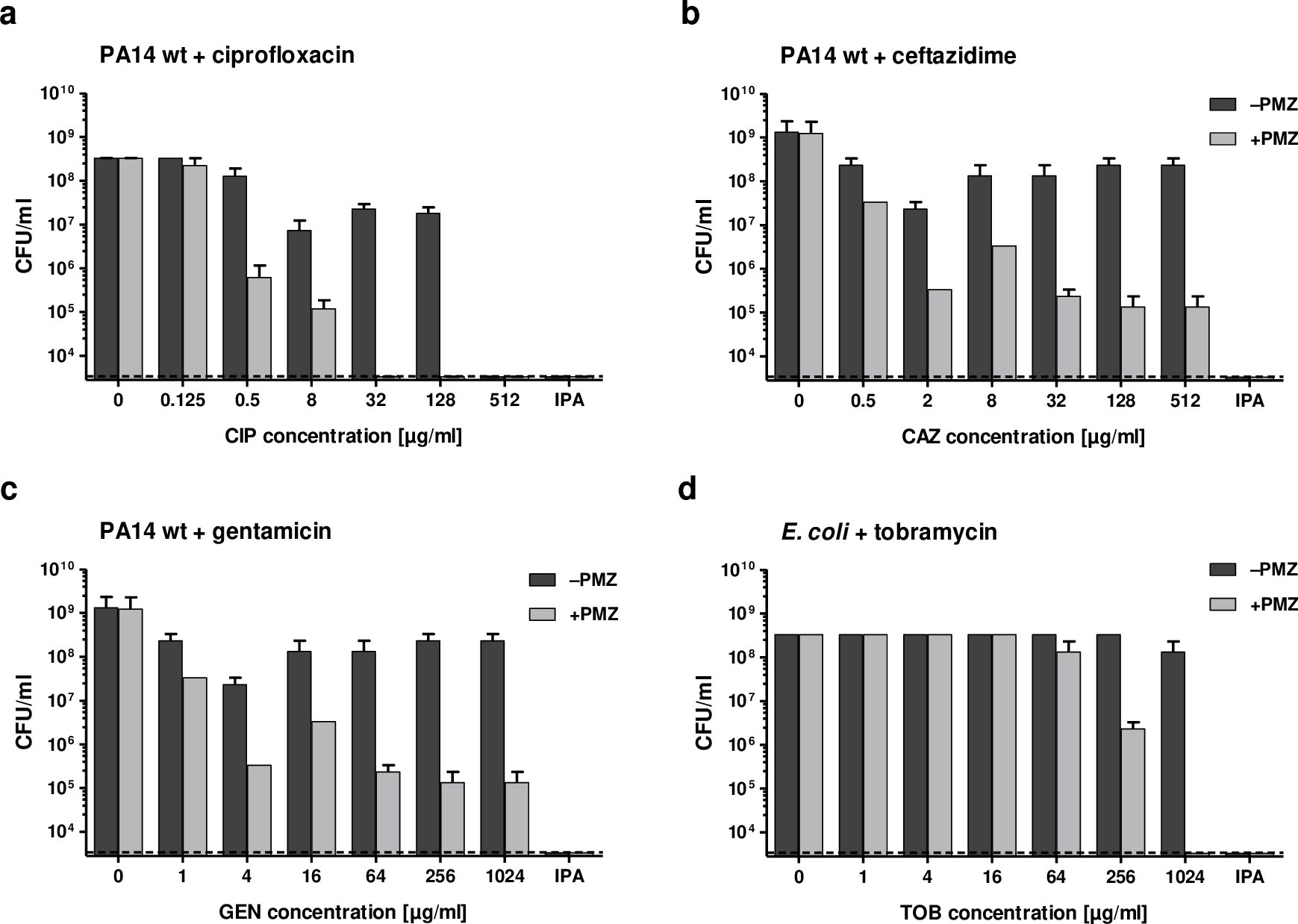

Supplement: S1 Fig — P. aeruginosa static biofilms grown in 96-well plates were treated with increasing concentrations of ciprofloxacin (CIP) (a), ceftazidime (CAZ) (b) or gentamicin (GEN) (c) with or without the addition of 100 μM promethazine (PMZ). Colony forming units (CFU) were determined from the antibiotics treated (dark grey) and antibiotics plus PMZ treated (light grey) biofilms. Isopropanol treatment (IPA) was used as killing control. Mean CFU counts were performed on pools of three wells in at least three independent experiments. Error bars represent the standard error of the mean, while the dashed line indicates the lower limit of detection of the assay. (d) Biofilms of Escherichia coli K12 were treated in the same manner with increasing concentrations of either tobramycin alone or in combination with promethazine, and CFU were determined accordingly. (TIF) [file ppat.1009126.s002.tif]

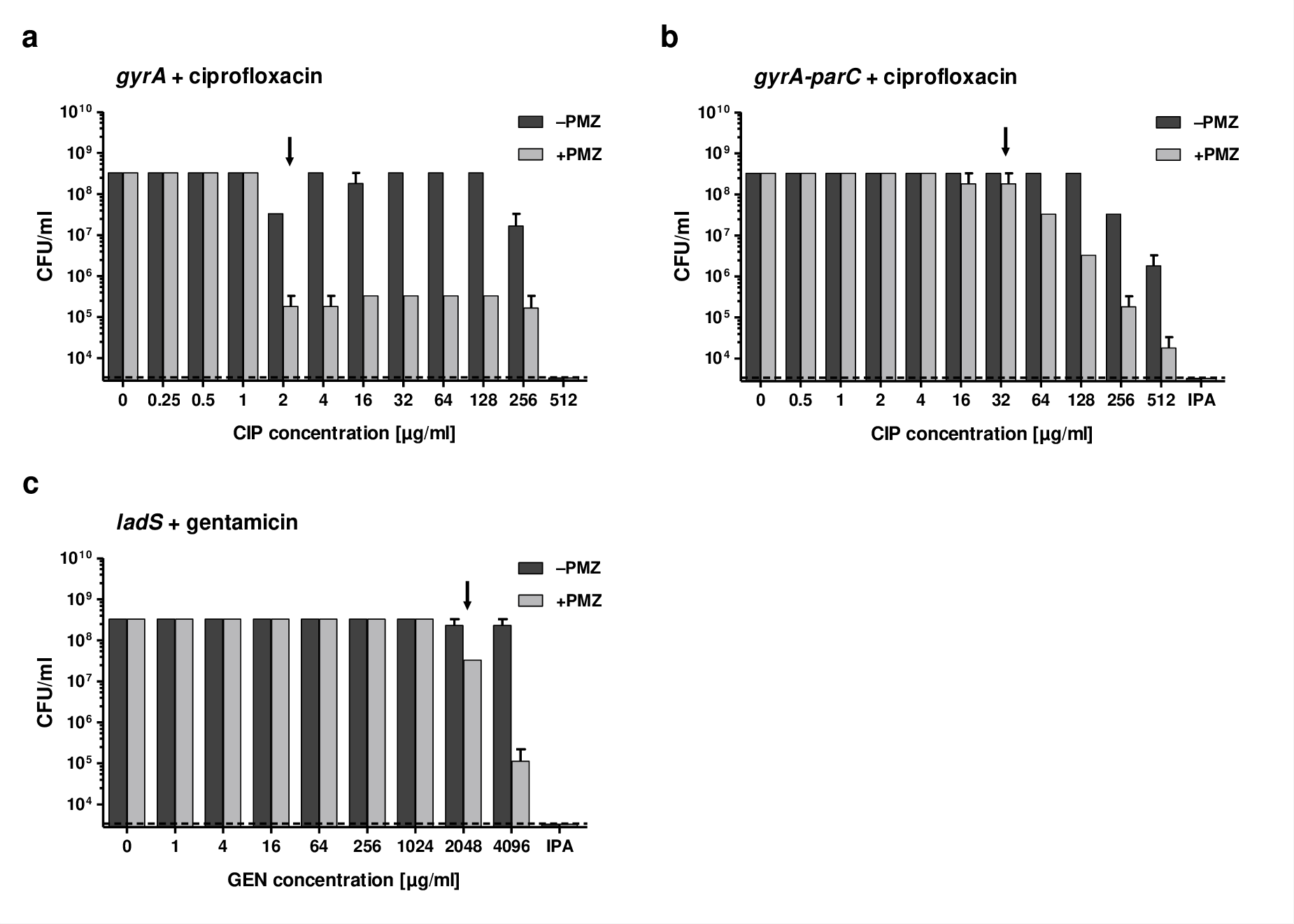

Supplement: S2 Fig — P. aeruginosa PA14 isogenic mutants, which exhibited an increase in the minimal inhibitory concentration (MIC indicated by the arrow) against ciprofloxacin due to a target mutation in gyrA (a), gyrA and parC (b) and gentamicin due to a gentamicin harboring resistance cassette transposon insertion (c), were grown in 96-well plates and treated with increasing concentrations of ciprofloxacin (a, b) and gentamicin (c) with or without the addition of 100 μM promethazine (PMZ). Colony forming units (CFU) were determined from the antibiotics treated (dark grey) and antibiotics plus PMZ treated (light grey) biofilms. Isopropanol treatment (IPA) was used as a killing control. Mean CFU counts were performed on pools of three wells in at least three independent experiments. Error bars represent the standard error of the mean, while the dashed line indicates the lower limit of detection of the assay. (TIF) [file ppat.1009126.s003.tif]

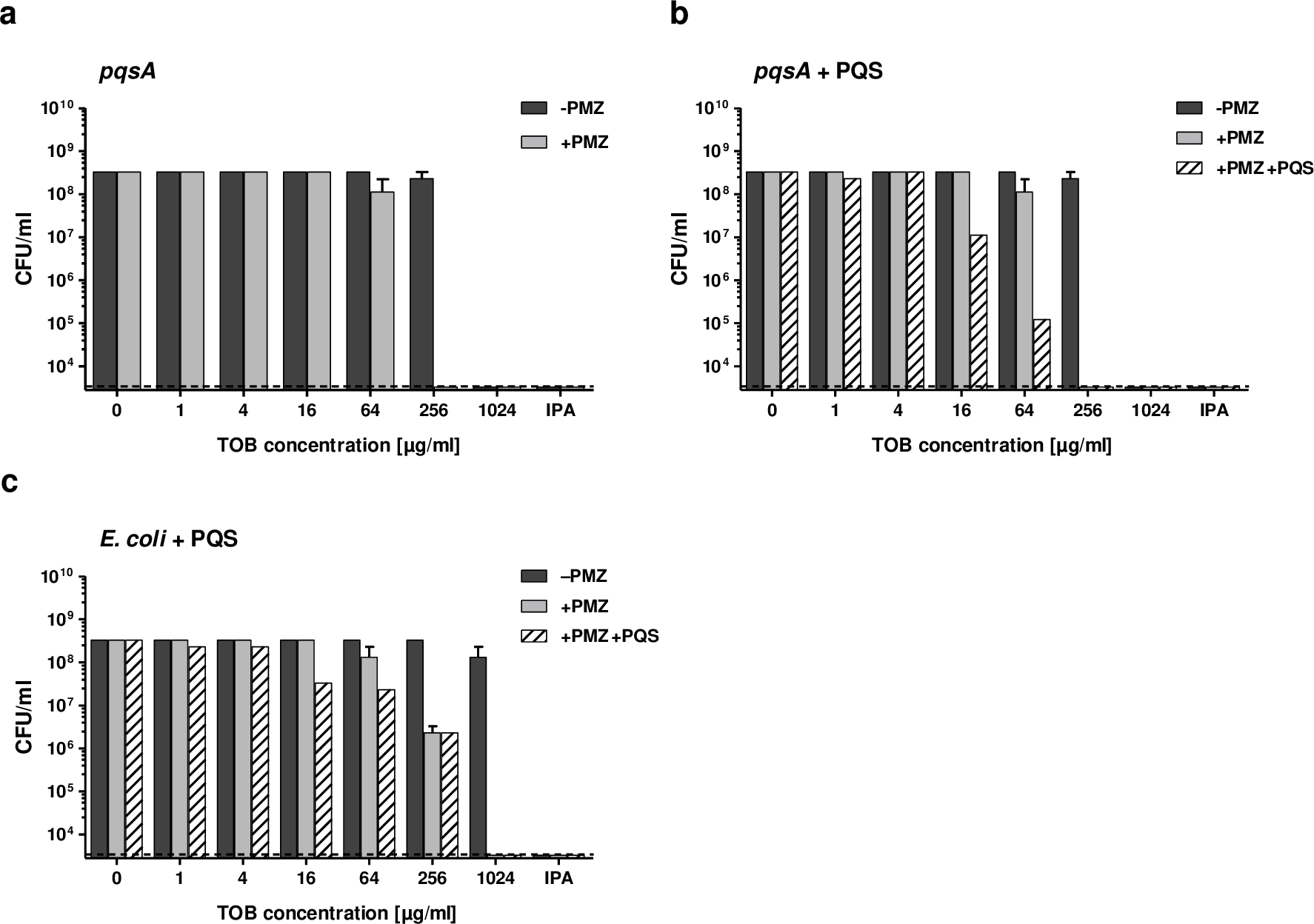

Supplement: S3 Fig — (a) A P. aeruginosa PA14 pqsA transposon mutant was grown in 96-well plates and treated with increasing concentrations of tobramycin with or without the addition of 100 μM promethazine (PMZ). Colony forming units (CFU) were determined from the tobramycin treated (dark grey) and tobramycin plus PMZ treated (light grey) biofilms. Isopropanol treatment (IPA) was used as a killing control. External addition of PQS (100 μM) restored the killing activity of tobramycin/PMZ in the P. aeruginosa PA14 pqsA transposon mutant (b) and enhanced the killing activity of tobramycin/PMZ in E. coli K12 (c). Mean CFU counts were performed on pools of three wells in at least three independent experiments. Error bars represent the standard error of the mean, while the dashed line indicates the lower limit of detection of the assay. (TIF) [file ppat.1009126.s004.tif]
